# Early Cardiopulmonary Fitness after Heart Transplantation as a Determinant of Post-Transplant Survival

**DOI:** 10.3390/jcm12010366

**Published:** 2023-01-03

**Authors:** Thomas C. Hanff, Yuhui Zhang, Robert S. Zhang, Michael V. Genuardi, Maria Molina, Rhondalyn C. McLean, Jeremy A. Mazurek, Monique S. Tanna, Joyce W. Wald, Pavan Atluri, Michael A. Acker, Lee R. Goldberg, Payman Zamani, Edo Y. Birati

**Affiliations:** 1 Division of Cardiology, Department of Medicine, Perelman School of Medicine, University of Pennsylvania, Philadelphia, PA 19104, USA; 2 Fuwai Hospital, Peking Union Medical College, Beijing 100006, China; 3 Division of Cardiovascular Medicine, NYU Langone Health, New York, NY 10016, USA; 4 Division of Cardiovascular Surgery, Department of Surgery, Perelman School of Medicine, University of Pennsylvania, Philadelphia, PA 19104, USA; 5 The Lydia and Carol Kittner, Lea and Banjamin Davidai Division of Cardiovascular Medicine and Surgery, Padeh-Poriya Medical Center, Azrieli Faculty of Medicine, Bar-Ilan University, Ramat Gan 5290002, Israel

**Keywords:** transplant, exercise, survival, prognosis

## Abstract

Background: Decreased peak oxygen consumption during exercise (peak Vo_2_) is a well-established prognostic marker for mortality in ambulatory heart failure. After heart transplantation, the utility of peak Vo_2_ as a marker of post-transplant survival is not well established. Methods and Results: We performed a retrospective analysis of adult heart transplant recipients at the Hospital of the University of Pennsylvania who underwent cardiopulmonary exercise testing within a year of transplant between the years 2000 to 2011. Using time-to-event models, we analyzed the hazard of mortality over nearly two decades of follow-up as a function of post-transplant percent predicted peak Vo_2_ (%Vo_2_). A total of 235 patients met inclusion criteria. The median post-transplant %Vo_2_ was 49% (IQR 42 to 60). Each standard deviation (±14%) increase in %Vo_2_ was associated with a 32% decrease in mortality in adjusted models (HR 0.68, 95% CI 0.53 to 0.87, *p* = 0.002). A %Vo_2_ below 29%, 64% and 88% predicted less than 80% survival at 5, 10, and 15 years, respectively. Conclusions: Post-transplant peak Vo_2_ is a highly significant prognostic marker for long-term post-transplant survival. It remains to be seen whether decreased peak Vo_2_ post-transplant is modifiable as a target to improve post-transplant longevity.

## 1. Introduction

Diminished exercise capacity is a cardinal feature of advanced heart failure, and a strong association between decreased peak oxygen consumption (Vo_2_) and increased mortality has been recognized in ambulatory heart failure patients for decades [1,2]. Given its association with mortality, peak Vo_2_ is commonly used to estimate prognosis, and current practice guidelines recommend its use as a threshold for advanced heart failure therapies [3]. Mechanistically, however, the relationship between Vo_2_ and mortality in heart failure with reduced ejection fraction (HFrEF) is confounded by multiple convergent pathophysiologic processes resulting in the diminished consumption of oxygen. While it is plausible that decreased peak Vo_2_ in HFrEF reflects, at least in part, inadequate tissue oxygen delivery during exercise due to a reduced cardiac output [4], a dissociation between peak Vo_2_ and hemodynamic dysfunction has long been observed [5].

Hypothetically, if cardiac output were the main limitation barring further increases in peak Vo_2_ then peak Vo_2_ should increase dramatically after heart transplantation, and Vo_2_ would no longer distinguish risk for mortality. Yet, across the population of heart-transplant recipients, post-transplant Vo_2_ is highly heterogeneous [6,7]. Peak Vo_2_ only improves in a small number of individuals, and those with the lowest pre-transplant peak Vo_2_ often have the lowest post-transplant Vo_2_ [8]. The reasons for this and the usefulness of Vo_2_ as a marker of short- or long-term survival are not well understood. Significant extracardiac parameters may mediate the link between mortality and peak Vo_2_, and advanced heart failure therapies that treat cardiac failure may not directly influence these parameters, including mechanical circulatory support and heart transplantation.

Because multiple comorbidities factor into the peak Vo_2_, there may be significant information captured in this metric post-transplant, even after transplant. This information could be clinically useful, improving risk-stratification and shedding new light on causal mechanisms for patient decline and transplant outcomes, with the potential for intervention. In this study, we sought to (1) quantify the association between post-transplant peak Vo_2_ and mortality and (2) identify potential pathophysiologic processes that may explain this association.

## 2. Materials and Methods

The first and last authors (T.C.H. and E.Y.B.) had full access to all the data in the study and take responsibility for its integrity and the data analysis. Requests to access deidentified data from qualified researchers trained in human subject confidentiality protocols should be sent to the corresponding author. Unidentified Stata code for this analysis is available upon request.

### 2.1. Design and Participants

We performed a retrospective analysis of all adult heart transplant recipients who were transplanted from March 2000 to November 2011 at the Hospital of the University of Pennsylvania, excluding multiorgan recipients and patients undergoing cardiac re-transplantation. Survival status was observed through October 2019 using data merged from the Penn Transplant Institute. This era was selected due to a clinical protocol at the time in which all patients underwent a post-transplant cardiopulmonary exercise test (CPET) within one year of transplant if clinically able. Patients who underwent CPET outside of their first post-transplant year were excluded, as were four patients with missing peak Vo_2_. Baseline pre- and post-transplant clinical data were recorded from the electronic medical record, including demographics, pre- and post-transplant comorbidities up to the time of CPET, laboratory values at the time of CPET, post-transplant echocardiographic data up to one year post-CPET, the incidence of acute cellular rejection grade 2R or 3R or antibody mediated rejection prior to CPET, and the CPET results themselves. This study was approved by the University of Pennsylvania Institutional Review Board with a waiver of informed consent.

### 2.2. Cardiopulmonary Exercise Metrics

Data from CPET were acquired retrospectively from the electronic medical record. All patients performed one symptom-limited CPET within the first post-transplant year according to our standard clinical practice, using a treadmill with a low- or moderate-intensity ramped protocol until exhaustion, the onset of limiting symptoms, or the development of a contraindication to continued exercise. Breath-by-breath expired gas analysis was used to obtain continuous estimates of Vo_2_ and carbon dioxide production (Vco_2_) at peak exercise. Studies were interpreted by an advanced heart failure cardiologist.

Absolute peak Vo_2_ was determined as the highest 10-s averaged samples obtained during exercise. We estimated the % of predicted peak Vo_2_ using the Fitness Registry and the Importance of Exercise National Database (FRIEND) equation based on age, sex, and weight [9]. Peak Vo_2_ is conventionally standardizing for total body mass; however, because Vo_2_ is influenced primarily by muscle mass and can be reduced out of proportion to heart failure severity in obese subjects [10], we examined values of Vo_2_ indexed separately for total body mass in kilograms (peak Vo_2, body mass_) and estimated lean body mass (peak Vo_2, lean_). Lean body mass was estimated from weight, height, age, and ethnicity using a formula validated against dual-energy X-ray absorptiometry (i.e., DXA scan) in a sample of 14,000 US adults [11]. Heart rate, systolic blood pressure, and diastolic blood pressure were recorded at rest and at peak exercise. O_2_ pulse was calculated as the ratio of absolute peak Vo_2_ to peak heart rate.

The respiratory exchange ratio (RER) was determined from the ratio of Vco_2_ to Vo_2_ at the time of exhaustion. Maximal Volitional exercise was determined using a peak RER ≥ 1.0, based on guideline recommendation and application in recent large studies of exercise in HFrEF [12,13,14,15]. Only studies demonstrating evidence of maximal effort were used in analyses. The first post-transplant transthoracic echocardiogram obtained up to one year after CPET was used to compare peak Vo_2_ to the left ventricular ejection fraction.

### 2.3. Statistical Analysis

Continuous data are expressed as mean ± standard deviation (SD), while categorical data are expressed as frequency and proportions. We compared baseline characteristics between patients who had peak Vo_2_ values above and below the sample median using the two-sample *t*-test for continuous variables (irrespective of normality due to large sample size) and the Chi-squared test or Fisher’s exact test as appropriate for categorical variables. All time-to-event analyses considered the time to death or re-transplantation from the time of transplant, referred to hereafter as simply “mortality” given the low incidence of re-transplantation. Differences in mortality over time in relation to absolute peak Vo_2_, %Vo_2_, peak Vo_2, body mass_, peak Vo_2, lean_ and O_2_ pulse were assessed in univariate and multivariable models as described below.

Three Cox proportional hazard models were constructed for each Vo_2_ parameter to quantify its crude and adjusted association with mortality. Model 1 was unadjusted. Model 2 was adjusted for demographics including age, race/ethnicity, sex, and the number of days from transplant to CPET. Finally, model 3 was adjusted for model 2 variables plus pre-transplant pulmonary vascular resistance (PVR), type 2 diabetes, ischemic cardiomyopathy, peripheral vascular disease, chronic obstructive pulmonary disease and post-transplant hemoglobin (measured at the time of CPET) [16]. All independent variables were normalized as a Z-score to their underlying SD to allow direct comparison of relative effect size. The proportional hazard assumption for each model was tested with Schoenfeld residuals [17]. A two-sided Type I error rate of 0.05 was used, without adjustment for multiple comparisons. The rate of missing data for each covariate did not exceed 15%. Missing values for covariates were imputed via multiple imputation with chained equations.

Next, we modeled survival at 1, 5, 10 and 15 years post-transplant as a function of post-transplant %Vo_2_ using flexible parametric survival models [18]. The %Vo_2_ cutoffs for predicted survival below 80% are presented at 5, 10, and 15 years post-transplant. One-year survival was also modeled, but a cutoff for 80% survival at 1-year did not exist within the measured range of %Vo_2_. All analyses were performed in Stata version 16 (College Station, TX, USA: StataCorp LLC).

## 3. Results

### 3.1. Characteristics of the Sample Population

**Figure 1:**
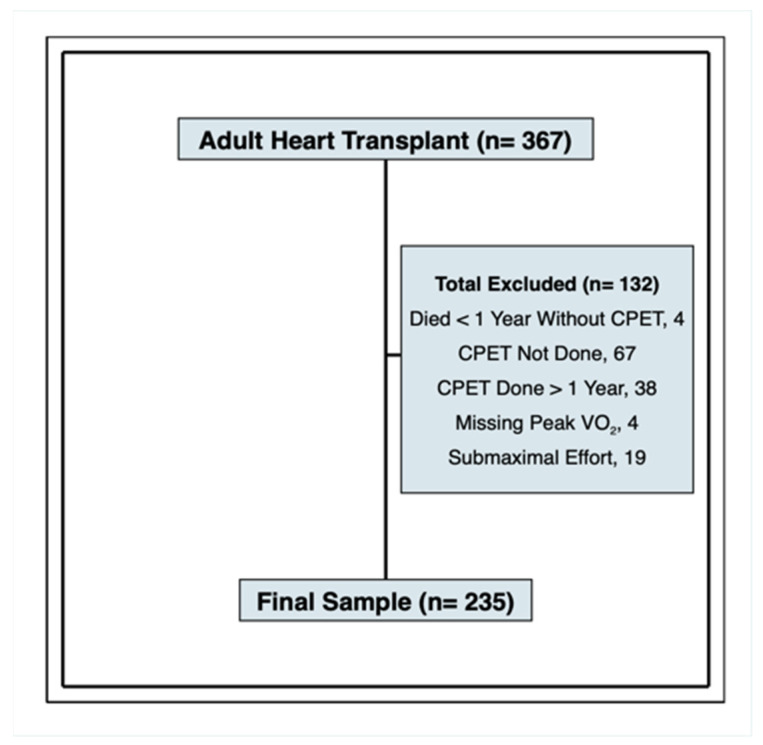
Study Inclusion and Exclusion. The final sample consists of 235 patients who had peak Vo_2_ measured within the first post-transplant year. Acronyms: CPET, cardiopulmonary exercise test; RER, respiratory exchange ratio.

A total of 367 patients underwent single-organ, first-time heart transplantation at the University of Pennsylvania during the study period (Figure 1). Of these, we obtained peak Vo_2_ measurements on 235 patients. Reasons for missing peak Vo_2_ measurements were: 4 died prior to one year without undergoing CPET, 38 performed CPET after the 1-year cutoff, 4 performed CPET but were missing peak Vo_2_, 67 were unable to perform CPET due to prolonged index hospitalizations or severe deconditioning, and 19 performed CPET with submaximal exercise. The RER for the patients excluded for submaximal exercise ranged from 0.8 to 1.0, while the RER for patients performing maximal exercise ranged from 1.0 to 1.44.

**Figure 2:**
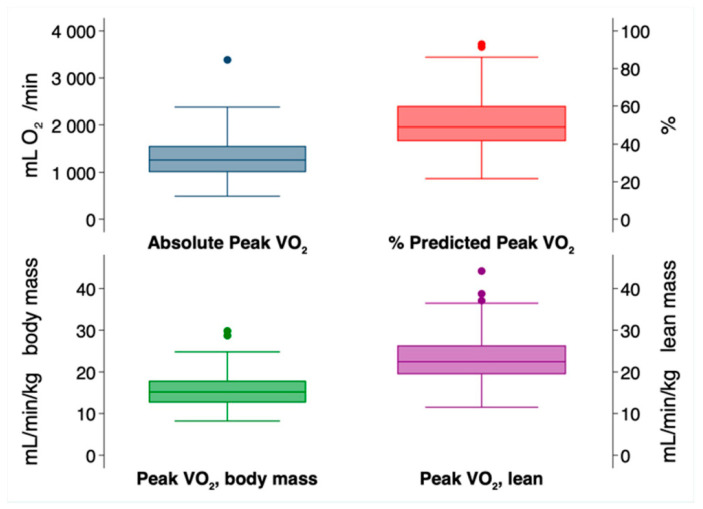
Distribution of Post-Transplant Peak Vo_2_ Indices. Box plots displaying the median, 1.5× interquartile range, and outliers for each Vo_2_ metric.

During follow-up of the 235 patients in the cohort, there were 93 deaths (40%) and 4 retransplants (1.7%). The range of follow-up was 115 days to 18.9 years (mean 10.5 years, SD ± 4.4 years). Figure 2 shows the distributions of post-transplant absolute peak Vo_2_, %Vo_2_, and Vo_2_ indexed to body mass or lean mass. The median %Vo_2_ was 49% (IQR 42 to 60%).

Several baseline parameters were associated with a %Vo_2_ below median (Table 1). This included younger age (50 vs. 56 years, *p* < 0.001), male sex (87 vs. 77%, *p* = 0.038), lower peak heart rate (116 vs. 125 bpm, *p* < 0.001), lower weight (82 vs. 88 kg, *p* = 0.013), and lower BMI (26 vs. 29 kg/m^2^, *p* < 0.001). Some of these relationships may seem paradoxical (i.e., younger age), but this is because younger patients tended to have lower peak Vo_2_ relative to their age-predicted values than older patients, in spite of having greater absolute peak Vo_2_ measurements. O_2_ pulse, the ratio of absolute Vo_2_ to peak heart rate, was also lower in individuals with %Vo_2_ below median (10 vs. 12 mL/beat, *p* < 0.001), suggesting impairments in the arteriovenous O_2_ content or stroke volume at peak exercise. Distributions of pre-transplant comorbidities and ethnicity were similar. No differences in post-transplant LVEF were observed between the groups (64% vs. 66%, *p* = 0.34), and there was no association between acute cellular rejection and %Vo_2_ (35% vs. 35%, *p* = 0.97). No patients who performed maximal exercise CPET had experienced antibody mediated rejection prior to CPET.

### 3.2. Association of Vo_2_ Metrics with Post-Transplant Mortality

**Figure 3:**
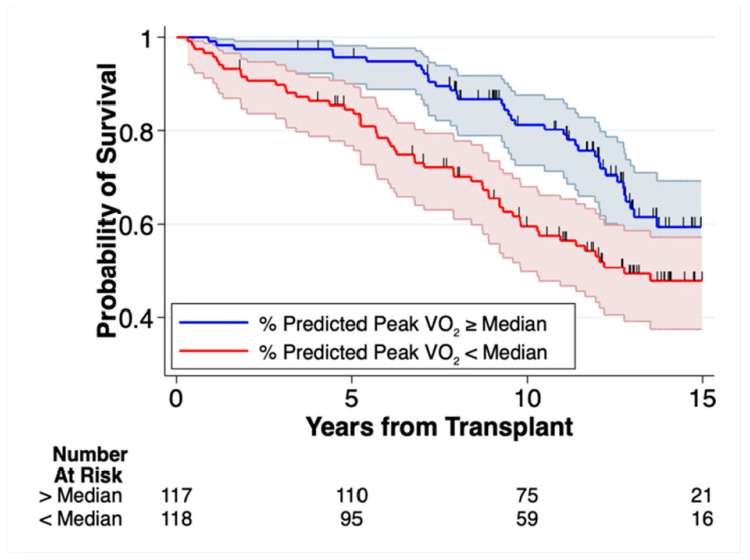
Kaplan–Meier Survival Estimates Stratified by Median % Predicted Peak Vo_2_. Patients with post-transplant % Predicted Peak Vo_2_ (%Vo_2_) below median had significantly lower long-term survival. The median %Vo_2_ was 49%. Vertical dash marks indicate censored events. Shaded areas represent the 95% Confidence Intervals.

All metrics of post-transplant peak Vo_2_ were significantly associated with mortality in unadjusted and multivariable adjusted models, with a large effect size (Table 2). Estimates for the effect of different peak Vo_2_ metrics on mortality were only marginally different. In univariate models, each SD increase in %Vo_2_ (14%) was associated with a 34% decrease in mortality (HR 0.66, 95% CI 0.53 to 0.84, *p* = 0.001), equivalent to a 2.4% decrease in mortality per each %Vo_2_. After adjusting for demographics, comorbidities, and the time from transplant to CPET (i.e., Model 3)—each SD increase in %Vo_2_ was associated with a 32% decrease in mortality (HR 0.68, 95 CI 0.53 to 0.87, *p* < 0.002), equating to a 2.3% decrease in mortality per %Vo_2_. We analyzed the association between peak Vo_2_ and mortality using alternative weight indices, including unindexed estimates (absolute peak Vo_2_) and peak Vo_2_ indexed to total body mass (Vo_2_, body mass) or estimated lean mass (peak Vo_2_, lean). All of these had estimates comparable to that of the %Vo_2_ (Table 2). Lastly, we evaluated whether the observed association between %Vo_2_ and mortality was conditional on heart rate by looking at the association of O_2_ pulse with mortality. The O_2_ pulse, in which the absolute peak Vo_2_ is indexed to the peak heart rate, demonstrated an equally strong association with mortality as other peak Vo_2_ indices (Table 2). In unadjusted models, each SD increase in the O_2_ pulse (3.0 mL O_2_/beat) was associated with a 19% decrease in the hazard of mortality (HR 0.81, 95% CI 0.65 to 0.99, *p* = 0.043). After multivariable adjustment, the HR for O2 pulse was 0.66 (95% CI 0.51 to 0.85, *p* = 0.002). Figure 3 displays the unadjusted Kaplan–Meier survival probability over time stratified by the median %Vo_2_ of 49%.

### 3.3. Predicting Post-Transplant Survival

**Figure 4:**
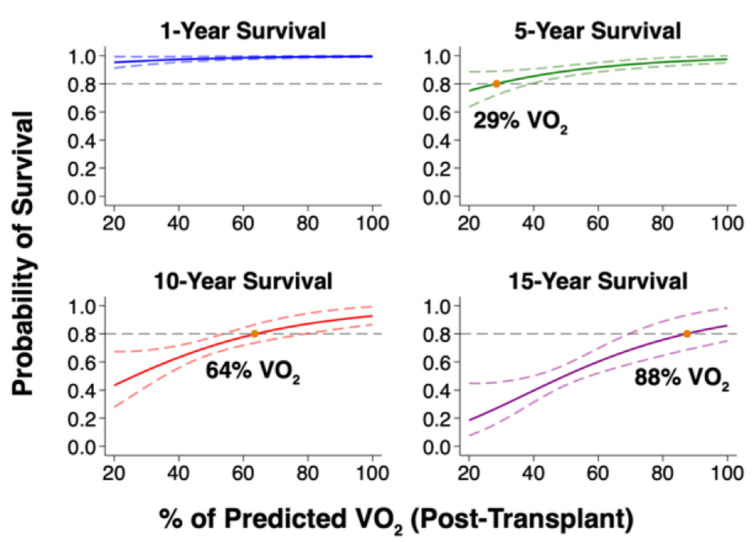
Predicted Survival Free from Re-transplantation based on Post-Transplant % Predicted Peak Vo_2_. Post-Transplant % Predicted Peak Vo_2_ predicts long-term survival. Plots based on unadjusted flexible parametric survival models, displaying mean survival (solid lines) along with upper and lower 95% confidence intervals (dashed lines). Acronyms: Vo_2_, oxygen consumption.

We derived four models predicting survival at 1, 5, 10 and 15 years post-transplant as a function of post-transplant %Vo_2_ (Figure 4). From each model, we identified a %Vo_2_ cutoff below which the predicted survival to each timepoint was less than 80%. At 5 years post-transplant, 80% survival (95% CI 72 to 89%) was predicted by a %Vo_2_ below 29%. At 10 years, the 80% survival threshold (95% CI 74 to 87%) was predicted by a %Vo_2_ below 64%. Finally, at 15 years, 80% survival (95% CI 69 to 94%) was predicted by a %Vo_2_ below 88%. Only 5 patients in our sample died within the first year (2%), so no cutoff predicting 1-year survival less than 80% could be estimated.

## 4. Discussion

An association between pre-transplant peak Vo_2_ and mortality in ambulatory heart failure was first established over two decades ago [1], and numerous studies have corroborated this association across multiple populations [2,19,20]. In this retrospective cohort analysis, we evaluated whether lower peak Vo_2_ in the early *post-transplant* period is associated with long-term post-transplant mortality. Our major findings were that: (1) diminished post-transplant peak Vo_2_ was strongly associated with mortality over nearly two decades of follow-up and (2) this association was independent of parameters known to directly influence Vo_2_, such as hemoglobin, sex, heart rate, body mass, and the number of days from transplant to CPET.

The etiology of diminished peak Vo_2_ is multifactorial, inVOlving cardiac and extracardiac parameters. Only some of these will respond directly to improved cardiac function, while some deficits are likely to remain, explaining why several studies have shown little improvement in peak Vo_2_ after heart transplant or mechanical circulatory support [6,8]. In our analysis, we attempted to differentiate whether the association between diminished peak Vo_2_ and mortality post-transplant was inherent to peak Vo_2_ or instead confounded by several common comorbidities. In the fully adjusted model, each SD (14%) increase in %Vo_2_ remained associated with a 32% decrease in the hazard of mortality (HR 0.68, 95 CI 0.53 to 0.87, *p* = 0.002). This effect size is in accordance with another single-center study out of Norway that analyzed transplants from the decade prior [21], confirming the generalizability of this association across time and institution. Moreover, we showed that this effect was independent of several additional extracardiac factors such as COPD, diabetes, peripheral vascular disease, and pulmonary vascular resistance.

These latter comorbidities, though not severe enough to preclude transplant in the first place, could still significantly confound the relationship between Vo_2_ and mortality. Instead, their adjustment had negligible effect. Similarly, the association between Vo_2_ and mortality was independent of post-transplant factors that can significantly impact the peak Vo_2_ or mortality, including body mass, LVEF, hemoglobin, creatinine, a history of acute cellular or antibody mediated rejection, the number of days from transplant to CPET, and immunosuppressive regimen. Additionally, the O_2_ pulse—which indexes the peak Vo_2_ to peak heart rate—was strongly associated with mortality in all multivariable models. This confirms that the association was not conditional on heart rate response, which can be diminished post-transplant secondary to sympathetic denervation. Based on the strong association between peak Vo_2_ and survival, higher %Vo_2_ predicted a greater probability of survival each year post-transplant. Using 80% survival probability as a target, this threshold was predicted at 5, 10 and 15 years by increasing %Vo_2_ cutoffs of 29%, 64%, and 88%, respectively. If externally validated, this metric could provide a useful prognostic screening tool for the early post-transplant period.

Diminished peak Vo_2_ that is unrelated to cardiac dysfunction or major comorbidities may indicate impaired skeletal muscle function, consistent with the “muscle hypothesis” in chronic heart failure [22]. Even in a healthy population, diminished exercise capacity is associated with increased mortality [23]; even more so in a heart failure population with deranged skeletal muscle oxidative capacity. Inadequate skeletal muscle oxidative capacity during exercise appears as a common element in multiple heart failure phenotypes, including heart failure with either reduced or preserved ejection fraction [24]. In chronic heart failure, this may be related to reduced O_2_ diffusion or deficiencies in mitochondrial density and efficiency [25], and these deficiencies post-transplant may be a holdover from the pre-transplant period. This is corroborated by the observation that lower pre-transplant peak Vo_2_ predicts lower post-transplant peak Vo_2_ [8], although skeletal muscle oxidative capacity could also worsen post-transplant secondary to chronic exposure to steroids and calcineurin inhibitors or prolonged hospitalizations [25]. Furthermore, it may be possible to detect reduced skeletal muscle oxidative capacity via a decline in the arteriovenous oxygen difference, implying decreased extraction and utilization of oxygen. Thus, it is notable that mortality in this study was strongly associated with decreased O_2_ pulse, which in addition to correlating with stroke volume also provides an indirect measure of the arteriovenous oxygen difference [26].

## 5. Limitations of Our Study

This study is retrospective in nature, which increases the possibility for misclassification bias using variables measured for clinical intent. In particular, peak Vo_2_ were determined via a 10 s average rather than 30 s, which may overestimate peak Vo_2_ (although this was measured in similar fashion for all subjects). As a single center study with a predominantly white population, generalizability to other sites may be reduced. In this study, most patients did not have pre-transplant CPET to analyze the prognostic importance of the change in parameters from pre- to post-transplant—this is a topic of future interest. Selection bias likely exists in the ability to perform a CPET, per se, thus estimates of association with mortality in the CPET population likely underestimate mortality in the overall heart transplant population that includes patients who were unable to perform CPET. Some covariate data were missing in this study, but no variables had greater than 15% missingness, and we were able to minimize bias through multiple imputation of missing datapoints.

## 6. Conclusions

Post-transplant peak Vo_2_ during maximal exercise is strongly associated with long-term post-transplant survival. The independent association between peak Vo_2_ metrics and mortality was independent of several important confounders and factors that contribute directly to Vo_2_. We observed an association between O_2_ pulse and mortality, suggesting that diminished post-transplant skeletal muscle oxidative capacity is a link between decreased peak Vo_2_ and survival. High-intensity exercise conditioning after transplant can safely augment peak Vo_2_ [27], but future studies are needed to evaluate whether improved peak Vo_2_ would lead to improved survival. If so, more aggressive cardiopulmonary rehabilitation post-transplant would be warranted, with peak Vo_2_ as an objective target.

## Figures and Tables

**Table 1:** Baseline Characteristics of Patients Above and Below Median % Predicted Vo_2_.

	% Predicted Peak Vo_2_, <Median of 48.6% *n* = 118	% Predicted Peak Vo_2_, ≥Median of 48.6% *n* = 117	*p*-Value
Baseline Characteristics			
Age at CPET, years	50 ± 13	56 ± (9)	<0.001
Sex, % male	103 (87%)	90 (77%)	0.038
Ethnicity			0.066
Non-Hispanic White	91 (77%)	95 (81%)	
Non-Hispanic Black	20 (17%)	22 (19%)	
Hispanic	4 (3%)	0 (0%)	
Other	3 (3%)	0 (0%)	
Diabetic Pre-Transplant	52 (44%)	43 (37%)	0.25
Ischemic Etiology Pre-Transplant	51 (43%)	47 (40%)	0.64
PVD Pre-Transplant	8 (8%)	3 (3%)	0.12
COPD Pre-Transplant	9 (9%)	8 (8%)	0.82
PVR Pre-Transplant, dynes * s * cm^−5^	146 ± 114	128.5 ± 72	0.19
Serum Creatinine (mg/dL)	1.6 ± 0.9	1.5 ± 1.0	0.39
Daily Prednisone Dose, mg	3.5 ± 3.5	3.1 ± 3.0	0.40
Cyclosporine Use	18 (15.3%)	16 (13.8%)	0.75
CPET Parameters			
Days from Transplant to CPET	88 ± 70	103 ± 79	0.12
Absolute Peak Vo_2_, mL O_2_/min	1095 ± 283	1528 ± 412	<0.001
% Predicted Peak Vo_2_	40 ± 6	62 ± 10	<0.001
O_2_ pulse, mL/beat	10 ± 3	12 ± 3	<0.001
Total Exercise Time, min	8 ± 3	9 ± 3	0.11
Peak Heart Rate, bpm	116 ± 17	125 ± 17	<0.001
Peak SBP, mmHg	149 ± 22	153 ± 22	0.18
Peak DBP, mmHg	81 ± 12	82 ± 11	0.71
Peak Respiratory Exchange Ratio	1.1 ± 0.1	1.1 ± 0.1	0.71
Hemoglobin at CPET, g/dL	13 ± 2	13 ± 2	0.23
Height, cm	176 ± 10	175 ± 10	0.35
Body Weight, kg	82 ± 19	88 ± 17	0.013
BMI at CPET, kg/m^2^	26 ± 5	29 ± 5	<0.001
Beta Blocker Use at CPET	25 (21%)	20 (17%)	0.46
Acute Cellular Rejection, %	35 (29.7%)	35 (29.9%)	0.97
Post-Transplant LVEF, %	64 ± 12	66 ± 7	0.34

Values are mean ± standard deviation or number (proportion). Acronyms: BMI, body mass index; COPD, chronic obstructive pulmonary disease; CPET, cardiopulmonary exercise test; DBP, diastolic blood pressure; SBP, systolic blood pressure; PVD, peripheral vascular disease; PVR, pulmonary vascular resistance; Vco_2_, carbon dioxide output; Vo_2_, oxygen consumption.

**Table 2:** Hazard Associated with each Standard Deviation Change in Peak Vo_2_.

	Model 1 Univariate		Model 2 Demographics		Model 3 Clinical Covariates	
	HR ^§^ (95% CI)	*p*-Value	HR (95% CI)	*p*-Value	HR (95% CI)	*p*-Value
Peak Oxygen Consumption Absolute Peak Vo_2_ (SD ± 414 mL O_2_/min)	0.75 (0.60, 0.93)	0.010	0.60 (0.46, 0.78)	0.000	0.62 (0.47, 0.81)	0.001
% Predicted Peak Vo_2_ (SD ± 14%)	0.66 (0.53, 0.84)	0.001	0.63 (0.50, 0.81)	0.000	0.68 (0.53, 0.87)	0.002
Peak Vo_2, body mass_ (SD ± 4 mL O_2_/min/kg body mass)	0.69 (0.55, 0.87)	0.002	0.66 (0.52, 0.83)	0.000	0.72 (0.56, 0.91)	0.007
Peak Vo_2, lean_ (SD ± 6 mL/min/kg lean mass)	0.62 (0.49, 0.78)	0.000	0.63 (0.50, 0.79)	0.000	0.68 (0.53, 0.86)	0.002
O_2_ Pulse (SD ± 3 mL O_2_/beat)	0.81 (0.65, 0.99)	0.043	0.66 (0.52, 0.85)	0.001	0.66 (0.51, 0.85)	0.002

Higher Peak Vo_2_ is associated with a decreased hazard of death or retransplantation. *Model 1*—unadjusted Cox proportional hazard model. *Model 2*—adjusted for age, race/ethnicity, sex, and days from transplant to CPET. *Model 3*—adjusted for Model 2 variables plus pulmonary vascular resistance, type 2 diabetes, ischemic cardiomyopathy, peripheral-vascular disease, chronic obstructive pulmonary disease, and hemoglobin. Hazard ratios (HR) are for a one standard deviation (SD) change in each metric. Acronyms: Vo_2_, oxygen consumption.

## Data Availability

The data that support the findings are available on request from the first author, T.C.H.

## Abbreviations

CPET	cardiopulmonary exercise test
HFrEF	heart failure with reduced ejection fraction
Peak Vo_2_	peak oxygen consumption
Peak Vo_2, body mass_	peak oxygen consumption indexed to total body mass
Peak Vo_2, lean_	peak oxygen consumption indexed to estimated lean body mass
RER	respiratory exchange ratio
%Vo_2_	percent of predicted peak oxygen consumption
VT	ventilatory threshold
